# Estimating the Incidence of Acute Infectious Intestinal Disease in the Community in the UK: A Retrospective Telephone Survey

**DOI:** 10.1371/journal.pone.0146171

**Published:** 2016-01-25

**Authors:** Laura Viviani, Mike van der Es, Lisa Irvine, Clarence C. Tam, Laura C. Rodrigues, Kathryn A. Jackson, Sarah J. O’Brien, Paul R. Hunter, IID2 Study Executive Committee

**Affiliations:** 1 Department of Infectious Disease Epidemiology, London School of Hygiene and Tropical Medicine, London, United Kingdom; 2 The Norwich School of Medicine, University of East Anglia, Norwich, United Kingdom; 3 Institute of Infection and Global Health, Liverpool University, Liverpool, United Kingdom; The University of Tokyo, JAPAN

## Abstract

**Objectives:**

To estimate the burden of intestinal infectious disease (IID) in the UK and determine whether disease burden estimations using a retrospective study design differ from those using a prospective study design.

**Design/Setting:**

A retrospective telephone survey undertaken in each of the four countries comprising the United Kingdom. Participants were randomly asked about illness either in the past 7 or 28 days.

**Participants:**

14,813 individuals for all of whom we had a legible recording of their agreement to participate

**Outcomes:**

Self-reported IID, defined as loose stools or clinically significant vomiting lasting less than two weeks, in the absence of a known non-infectious cause.

**Results:**

The rate of self-reported IID varied substantially depending on whether asked for illness in the previous 7 or 28 days. After standardising for age and sex, and adjusting for the number of interviews completed each month and the relative size of each UK country, the estimated rate of IID in the 7-day recall group was 1,530 cases per 1,000 person-years (95% CI: 1135–2113), while in the 28-day recall group it was 533 cases per 1,000 person-years (95% CI: 377–778). There was no significant variation in rates between the four countries. Rates in this study were also higher than in a related prospective study undertaken at the same time.

**Conclusions:**

The estimated burden of disease from IID varied dramatically depending on study design. Retrospective studies of IID give higher estimates of disease burden than prospective studies. Of retrospective studies longer recall periods give lower estimated rates than studies with short recall periods. Caution needs to be exercised when comparing studies of self-reported IID as small changes in study design or case definition can markedly affect estimated rates.

## Introduction

Infectious intestinal disease (IID), presenting as diarrhoea and vomiting, contributes a substantial disease burden even in high-income countries like the UK [1–4]. In five recently completed telephone surveys conducted across European Union Member States, self-reported illness rates ranged from 1.4 cases per person per year in Denmark to 0.33 cases per person per year in France [5–9]. However, comparing international rates is hampered by disparities in case definitions, study designs, periods of recall of symptoms and the characteristics of the populations studied [10,11]. Most people with IID do not seek medical attention [2,4], and even when they do their illness is often not investigated or reported [12], so it is difficult to determine accurately the population incidence of disease based on national surveillance or other routinely collected data.

In the UK two large burden of IID studies have been undertaken 15 years apart: IID1 from 1993–6 [4,13] and IID2 from 2008–9 [2,14]. Both studies incorporated a retrospective and a prospective component. Estimates of population burden of disease differ substantially between retrospective and prospective study designs even when using identical case definitions. This was highlighted in the IID1 Study, in which the incidence of IID estimated using a retrospective design was 0.55 episodes per person-year, compared with 0.19 per person-year in the prospective cohort component (FSA, 2000). Prospective cohort studies are prone to several biases, including loss to follow-up, and reporting fatigue whereas common issues with retrospective surveys include inaccurate recall, response bias and sampling bias. The majority of retrospective studies are based on a 28 day recall period, although it has been suggested recently that respondents’ recall accuracy declines with increasing time [15].

We present here for the first time the results from one component of the IID2 study: the telephone survey. The aim of this component was to estimate via a national telephone survey of self-reported diarrhoea and vomiting the rate of cases of IID in the population of each UK country, based on a 7 day and a 28 day recall period.

## Methods

The methods of the complete IID2 study and its telephone study component have been described in detail elsewhere [14]. In brief, for the telephone study component, we conducted a telephone survey of self-reported IID in the four UK countries between 1 February 2008 and 31 August 2009. Households were sampled by random digit dialling (RDD) of fixed landlines. We did not sample mobile telephone numbers because of difficulties in obtaining reliable geographical location, and because of ethical issues in potentially contacting mobile telephones in use by children. To obtain a set of valid telephone number stems for RDD, we selected the telephone numbers of 100 randomly chosen general practice (GP) clinics and deleted the last three digits. A pilot study conducted between September and December 2007 indicated that this method tended to oversample individuals in urban areas due to the greater concentration of GP clinics in inner cities. To overcome this, we added the telephone stems of the 21,750 primary schools across the UK to the telephone number database (following the observation that primary schools are more evenly spread throughout the territory than GP practices are), deleting any duplicate stems and deleting the last three digits as above.

We selected individual households by dialling a randomly selected telephone stem from the database and replacing the last three digits by a randomly generated number between 000 and 999. A team of six to ten trained telephonists made calls between 5pm and 9pm on weekdays and between 10am and 2pm at weekends. If the number dialled was invalid, belonged to a commercial property or a fax machine, or if a valid household refused to take part in the study, the number was not dialled again. For valid numbers, telephonists made up to three attempts to contact the household on different occasions.

To randomly select a participant from each household, telephonists asked to speak to the *n*th oldest resident in the household, *n* being a computer-generated random number based on the number of people at home at the time of the call. For example, if at the time of the call 3 people were present in the household, then the computer generated a random number between 1 and 3; let us suppose that the number 3 was generated, then the telephonist asked to speak to the youngest person (i.e. 3^rd^ oldest) present in the household at the time of the call. All participants gave oral consent to take part in the survey. If the person selected was <12 years of age, the parent or guardian was asked to respond on their behalf. For participants between 12 and 16 years of age, the parent or guardian could choose to allow the child to be interviewed directly or to respond on their behalf.

The survey incorporated questions on socio-demographic characteristics, and recent history of diarrhoea and/or vomiting. Those reporting symptoms were asked additional questions about the duration and severity of symptoms, use of healthcare services and recent travel outside the UK. To investigate whether the frequency of symptom reporting varied according to recall period, we assigned participants randomly to two groups. The first was asked to recall IID symptoms within the previous seven days (80% of interviews) or 28 days (20% of interviews). Telephonists entered data directly onto a bespoke, secure, Microsoft Access™ database during the course of the interview. Calls were also recorded using CopyCall Telephone Recorder or Retell 957 software, to allow double data entry for data validation, and to fulfil the ethical requirement for documented informed consent.

The prospective component of the IID2 study has also been described in detail elsewhere [14], but we summarise here the main aspects. The study was conducted over the same period as the telephone survey, and the same definition of IID was used. 6836 participants from 88 GP practices were asked to report symptoms of diarrhoea and/or vomiting on a weekly basis, either by e-mail or prepaid postcard for up to 52 weeks. The participants reporting symptoms were subsequently asked to complete a questionnaire investigating the type and the duration of symptoms, recent travel outside the UK, the use of healthcare services, and they were asked to submit a stool specimen for testing.

### Sample size

The sample size calculations were based on an expected frequency of self-reported IID of 6% (95% confidence interval (CI): 4%–8%) over a 28-day period, and 1.5% (95% CI: 1%–2%) over a 7-day period [4]. The required numbers of completed interviews were approximately 2,000 for the 28-day recall group and 10,000 for the seven-day recall group. The 5:1 ratio of calls between the seven-day and 28-day recall groups was designed to provide a comparable number of person-days of recall in the two groups, on the assumption that frequency of reported symptoms was independent of recall period, thus allowing for a similar level of precision in the two estimates. The interviews were split evenly between the four UK countries to enable similar levels of precision of estimates in each country for between-country comparisons. We increased the required sample size in each country by 20% to allow for possible differentials in participation between countries, resulting in 2,400 and 12,000 calls in the 28-day and seven-day recall groups.

### Case definition

The primary case definition was that used in the original IID study [4]. Cases of IID were defined as people with loose stools or clinically significant vomiting lasting less than two weeks, in the absence of a known non-infectious cause. Vomiting was considered clinically significant if it occurred more than once in a 24-hour period and if it incapacitated the case or was accompanied by other symptoms such as cramps or fever. We excluded symptomatic individuals with non-infectious causes of diarrhoea or vomiting, including Crohn’s disease, ulcerative colitis, cystic fibrosis, coeliac disease, surgical obstruction, excess alcohol, morning sickness and, in infants, regurgitation. We also excluded symptomatic individuals who reported having travelled outside the UK in the 10 days prior to illness onset. In addition to using the above case definition we also calculated incidence rates based on the World Health organization definition of three or more loose stools in a 24 hour period or vomiting [16].

### Data analysis

We calculated the incidence rate of self-reported IID as the number of cases of IID among survey participants divided by the total person-time of follow-up, separately for the 7-day and 28-day recall groups. We made two adjustments to account for the expected prevalence of non-infectious diarrhoea in the population and the proportion of time spent outside the UK. For each age and sex category, we obtained estimates of non-infectious diarrhoea prevalence from the proportion of individuals excluded in a concurrent population cohort study of IID. We obtained estimates of the average proportion of time spent outside the UK in each age group and sex category from the 2008 International Passenger Survey (http://discover.ukdataservice.ac.uk/Catalogue/?sn=5993&type=Data%20catalogue). The relevant fractions of person-time were then subtracted from the denominator.

We weighted the incidence estimates to adjust for differences in the age and sex distribution of participants relative to the 2001 census population of each country using post-stratification weighting [17], thereby giving greater weight to strata with proportionately fewer participants. Rate estimates were further adjusted to reflect the relative sizes of the populations in each UK country (England comprises 83.6% of the UK population, Scotland 8.6%, Wales 4.9% and Northern Ireland 2.9%). Finally, we adjusted for the number of interviews completed each month. This was done in order to avoid bias due to seasonal effects, because the number of interviews conducted varied by month. We used jackknife re-sampling methods [18] to calculate 95% confidence intervals.

### Ethics statement

All participants gave informed verbal consent which was recorded using CopyCall Telephone Recorder or Retell 957 software. The North West Research Ethics Committee approved this study (07/MRE08/5).

## Results

### Recruitment and representativeness

From 1^st^ February 2008 to 27^th^ August 2009, 78,878 telephone numbers were dialled across the four UK countries (Fig A in S1 File). Of these, 28,776 (36.5%) numbers were not eligible because they were invalid numbers (n = 24,341, 30.9%), commercial numbers (n = 4,395, 5.6%), or because the person answering the telephone did not speak English (n = 40, 0.05%). For 16,381 numbers (20.8%), it was not possible to ascertain whether the number dialled belonged to an eligible household, because the call was not answered (n = 10,222, 13%), it reached an answering machine (n = 3,693, 4.7%) or a fax machine (n = 2,108, 2.7%), or the number was engaged (n = 358, 0.4%). Thus, 33,721 (42.7%) numbers belonged to households eligible to take part in the survey.

Of the 33,721 eligible calls, 16,208 (48.1%) interviews were successfully completed, and similar completion proportions were observed by month of study (Fig B in S1 File) and between the two recall periods. The proportion of completed calls (Table A in S1 File) was similar in England (51.7%, 95% CI: 50.5%–52.8%), Scotland (49.9%, 95% CI: 48.8%–51.1%) and Wales (49.7%, 95% CI: 48.7%–50.7%) but was lower in Northern Ireland (41.7%, 95% CI: 40.7%–42.7%). Although the proportion of calls resulting in completed interviews was fairly constant over time, the number of interviews completed each month increased dramatically from January 2009, because more calls per month were achieved during this period as a result of increased staffing.

We restricted the analyses to the 14,813 calls for which evidence of consent was clearly recorded in the audio file: for 1,395 interviews the audio recording was missing or damaged. A further 87 calls were subsequently excluded from the analyses because the date of onset of symptoms was outside the period over which the participant was asked to recall. After exclusions, 14,726 interviews were available for analysis. Among survey participants, the survey respondent was randomly selected among the people present in the household at the time of the call, though if the randomised person was not prepared to participate we accepted to initial respondent. Full randomisation was obtained for 45.7% of calls in the 7-day recall group and for 45.2% of calls in the 4-week recall group.

**Fig 1:**
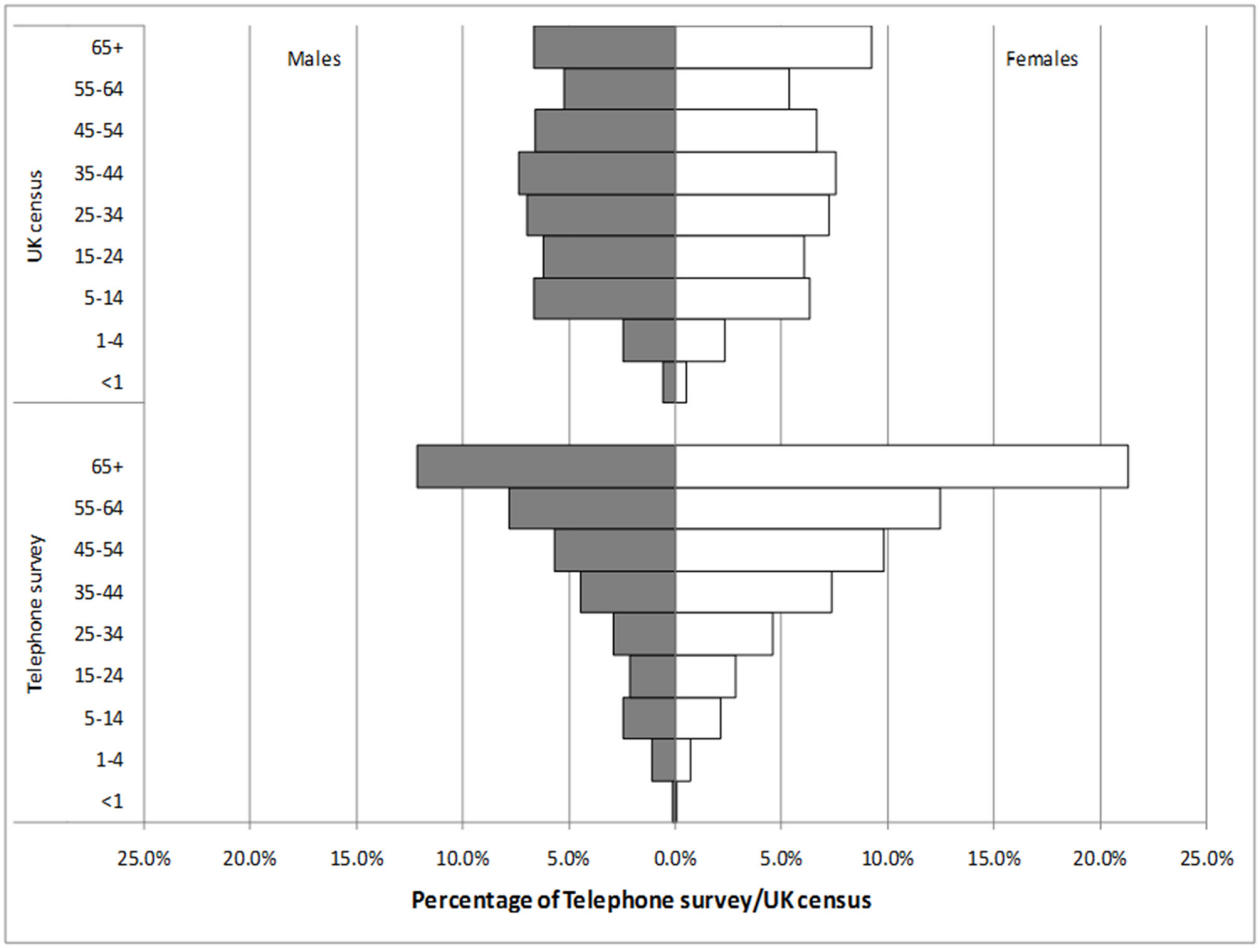
Age and sex structure of telephone survey participants compared with the UK census population.

Fig 1 compares the age and sex structure of participants in the telephone survey with the UK census population. Females and elderly participants were over-represented in the survey sample. The majority of telephone survey participants (96.4%) were of White ethnicity, while other ethnic groups were slightly under-represented relative to the UK census population (Fig C in S1 File). Survey participants were broadly representative of the UK population in terms of household size, although there was a small deficit of single-person households and a slight excess of two-person households in the study (Fig D in S1 File).

Individuals living in the most deprived areas were under-represented in the telephone survey: approximately 25% of survey participants lived in areas in the first two quintiles of area-level deprivation, compared with the expected 40% of the UK population (Fig E in S1 File). By contrast, individuals living in rural areas and towns were over-represented in the survey sample (Fig F in S1 File).

### Incidence rates in the telephone survey

The estimates of IID incidence in the telephone survey for the 7-day and 28-day recall groups are shown in Table 1. Among participants in the 7-day recall group, there were a total of 300 cases and 212 person-years, resulting in a crude incidence of IID of 1,414 cases per 1,000 person-years (95% CI: 1263–1583). Among the 28-day recall group, 107 cases occurred in 158 person-years, giving a crude incidence of IID of 676 cases per 1,000 person-years (95% CI: 559–817). After standardising for age and sex, and adjusting for the number of interviews completed each month and the relative size of each UK country, the estimated rate of IID in the 7-day recall group was 1,530 cases per 1,000 person-years (95% CI: 1135–2113), while in the 28-day recall group it was 533 cases per 1,000 person-years (95% CI: 377–778).

**Table 1:** Incidence rate of overall IID in the Telephone Survey by recall period.

			Crude rate		Adjusted rate			
Recall period	Cases	PY^a^	Rate^b^	(95% CI)	Rate^b^	(95% CI)	RR^c^	(95% CI)
**7 days**	300	212	**1414**	(1263–1583)	**1530**	(1135–2113)	**2.9**	(1.8–4.6)
**28 days**	107	158	**676**	(559–817)	**533**	(377–778)		

^a^PY—person-years;

^b^Cases per 1000 person-years;

^c^RR—Rate ratio comparing incidence in 7-day and 28-day recall groups.

Table 2 presents incidence estimates by age group and sex. Rates decreased with age in the 7-day recall period. For the 28-day recall period the pattern was less clear, but the number of cases identified in each age group was small.

**Table 2:** Incidence rate of overall IID in the Telephone Survey by recall period, age group and sex. Estimates are standardised by age and sex according to the UK census population and they are adjusted taking into account different size of the populations in each UK country, prevalence of non-infectious diarrhoea in the population, proportion of time spent outside the UK, and number of interviews completed each month.

	7-day recall			28-day recall			Rate ratio	
	PY^a^	Rate^b^	(95% CI)	PY^a^	Rate^b^	(95% CI)	RR^c^	(95% CI)
Age group								
<1 year	0.4	**---**^d^	---	0.4	**790**	(13–2670)	**---**	---
1–4 years	4.1	**2,910**	(1,218–8,534)	3.7	**336**	(130–977)	**8.7**	(2.4–31.1)
5–14 years	10.7	**2,020**	(538–12,986)	6.9	**1,037**	(389–3,463)	**1.9**	(0.4–8.5)
15–24 years	11.7	**1,194**	(556–3,016)	7.9	**60**	(23–191)	**20.0**	(5.9–67.8)
25–34 years	15.3	**2,177**	(1,025–5,467)	11.3	**292**	(51–4,051)	**7.5**	(1.6–35.8)
35–44 years	25.1	**1,369**	(828–2,426)	18.0	**809**	(375–2,022)	**1.7**	(0.7–4.3)
45–54 years	35.1	**1,633**	(958–3,014)	27.4	**726**	(347–1,775)	**2.2**	(0.9–5.6)
55–64 years	43.3	**799**	(505–1,343)	31.7	**764**	(340–2,069)	**1.0**	(0.4–2.7)
65+ years	66.4	**1,028**	(687–1,607)	51.0	**247**	(120–594)	**4.2**	(1.8–9.6)
Sex								
Males	81.8	**1,669**	(1,173–2,457)	60.4	**545**	(306–1,067)	**3.1**	(1.5–6.1)
Females	130.3	**1,401**	(846–2,497)	98.0	**523**	(346–822)	**2.7**	(1.4–5.1)

^a^PY—person-years;

^b^Cases per 1000 person-years, adjusted for number of interviews completed each month and the relative size of each UK country;

^c^Rate ratio comparing 7-days and 28-day recall groups;

^d^No cases reported.

Overall, the rate estimated in the 7-day recall group was approximately 3 times higher than that estimated in the 28-day recall group (Table 1). There was considerable variation by age: the rate ratios comparing incidence in the 7-day and 28-day recall groups were generally higher among those aged <35 years (Table 2). The fact that there were no reported cases in children under 1 year of age in the 7 day recall period study should not be over-interpreted due to very small numbers recruited. The rates in males and females were similar for both recall periods.

The rates by country are shown in Table 3. There was variation in the rates between countries for both recall periods. However, there was considerable overlap in the 95% CIs. There was no clear pattern in incidence by household size, area-level deprivation or urban-rural classification (data not shown). Incidence estimates were highest among participants living in households with 4 people. By contrast, participants living in rural areas reported the lowest rates of IID in the 7-day recall group, but the highest rates in the 28-day recall group. It should be noted, however, that there was considerable uncertainty around these rate estimates.

**Table 3:** Incidence rate of overall IID in the telephone survey by recall period and country. Estimates are standardised by age and sex according to the UK census population and they are adjusted taking into account different size of the populations in each UK country, prevalence of non-infectious diarrhoea in the population, proportion of time spent outside the UK, and number of interviews completed each month.

	7-day recall		28-day recall	
Country	Rate	(95% CI)	Rate	(95% CI)
England	**1,463**	(994–2,247)	**449**	(280–767)
Northern Ireland	**1,270**	(932–1,775)	**802**	(513–1325)
Scotland	**2,053**	(1,444–3,020)	**1,196**	(756–2,007)
Wales	**2,066**	(1,579–2,759)	**662**	(398–1,184)

**Fig 2:**
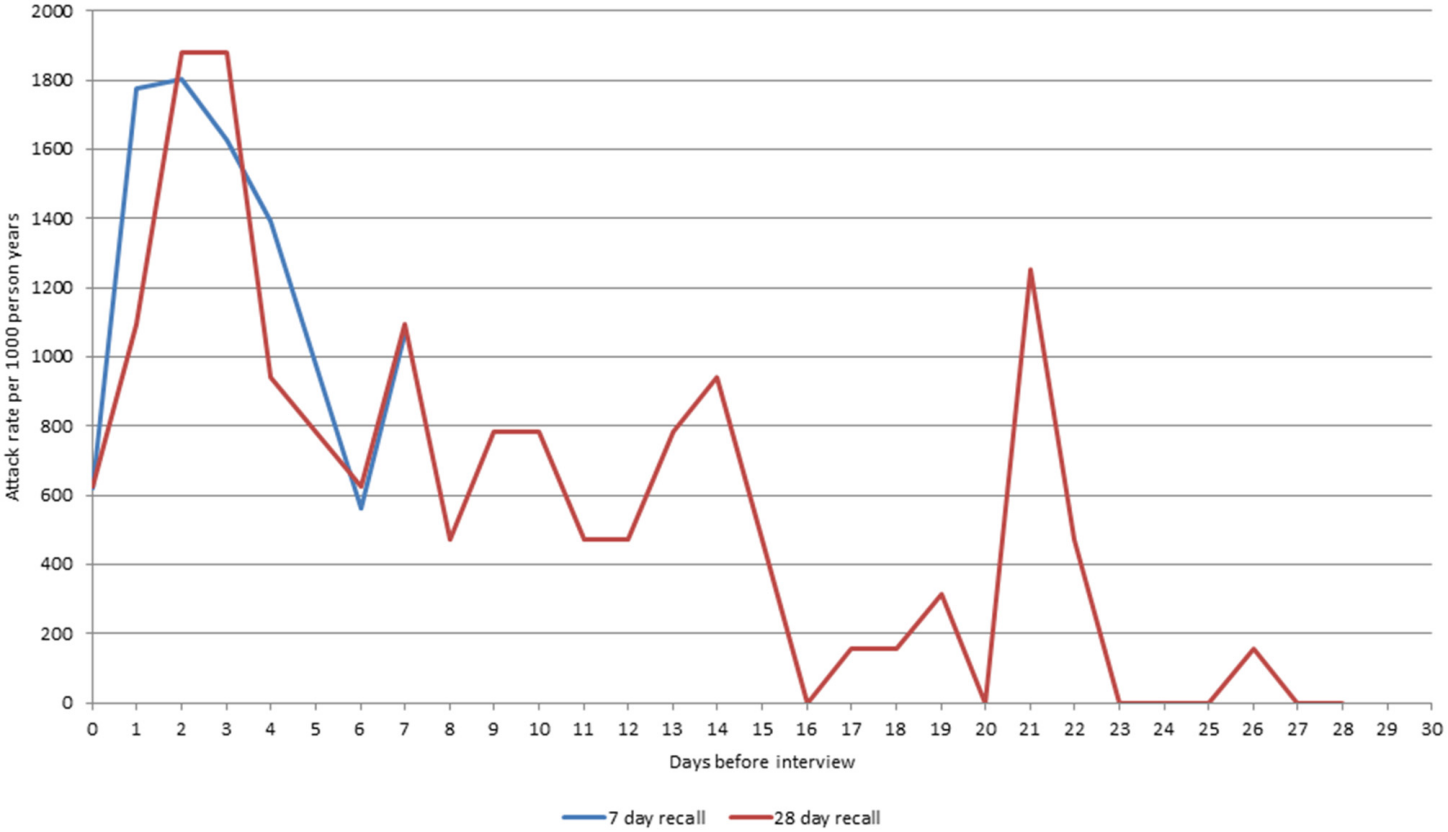
Decay in the reporting of symptoms among telephone survey participants by recall group^a^. ^a^Each data point represents the calculated crude annual incidence rate based on the number of participants reporting onset of symptoms on day of interview (x = 0) and each day prior to the date of interview.

For both the 7-day and 28-day recall, there was evidence of variation in recall of IID symptoms according to time since illness onset. Participants reported a higher number of episodes with onset in the 3 days prior to interview, but there was a rapid decline in the number of episodes reported with onset beyond this period (Fig 2). For the 28-day recall group, there was also clear evidence of digit preference, with a greater number of episodes reported with onset 7, 14 and 21 days prior to the date of interview than on other days.

When using the WHO definition of three or more episodes of diarrhoea then reported illness rates were approximately 20% lower than when using the IID2 definition. The crude rates with the international definition were 1,116 and 551 cases per 1,000 person years for 7 and 28 day recall periods. The equivalent adjusted rates were 1,327 and 509 cases per 1,000 person years.

When we compared the overall IID rates estimated with the telephone survey with the IID rates of the prospective component of the IID2 study, we found that they were remarkably higher, both for the 7-day and the 28-day recall periods: 1,530 cases per 1,000 person years (95%CI: 1,135; 2,113) and 533 cases per 1,000 person-years (95%CI: 377;778) vs 274 cases per 1000 person-years (95% CI 254 to 296).

## Discussion

The estimated rates from the telephone survey (28-day recall) were within the range reported in the international literature [5–9,11]. In particular the rate estimates in the 28-day recall telephone survey were very close to the rates reported in the retrospective component of the IID1 Study (533 per 1,000 person-years in IID2 versus 550 per 1,000 person-years in IID1) [4]. On the other hand, the results differ quite markedly from published prospective cohort studies in the UK (Wheeler *et al*. 1999; Tam *et al*. 2012) [2,4], the Netherlands [3] and the US [19].

Nearly 50% of individuals across UK invited to take part to the telephone survey completed a questionnaire, albeit with variation across countries. This level of participation is similar to telephone surveys from British Columbia (44%) [20], Canada (34.7%) and the United States (37.1%) but is lower than the ones achieved in Ireland (84.1%) and Australia (68.2%) [21]. However, a study examining three telephone surveys carried out on the island of Ireland, reported that participation fell from 84.1% to 40.5% between 2000 and 2005 [22]. This decline in participation might reflect a general attitude towards telephone surveys that might be echoed in our study, putting it in line with other studies in terms of level of participation.

People in the younger age groups were least likely to participate, especially young males. Younger people are more likely to use mobile phones but, mainly for ethical reasons, we were unable to make calls to mobile numbers. This might be one of the reasons why this group was under-represented in our study. However, among participants in the prospective cohort of the IID2 study, 95% still used a land line as their main method of making phone calls. This suggests that the potential for bias from exclusion of mobile telephones was small. In any case, to account for under-representation of males and of certain age groups, we standardised rates according to the age and sex distribution of the census population.

The other group that was substantially under-represented compared to the National census was ethnic minorities, but in this case we were unable to standardize for this group due to small numbers and complexity in the range of different ethnic minorities. Ethnic minorities represent about 10% of the UK population; unless they experience very different patterns of IID from the other ethnic groups, their under-representation in our study is not likely to to have a major impact on estimated rates.

Random sampling of people within the household proved difficult to implement. For both recall periods, in less than 50% of calls it was possible to randomly select the participant among the people present in the household. A similar pattern was seen in a prior telephone survey in Northern Ireland where the person who answered the call was most likely to complete the survey [23]. However, in our study, the rate estimates among those sampled at random and those not sampled at random were very similar (data not shown), which suggests that among those present in the household at the time of the call, the decision about who responded to the survey was not primarily influenced by whether participants recently had symptoms.

We did not define the term “diarrhoea” to participants. Most investigators who use telephone surveys to estimate illness burden define diarrhoea as three or more loose stools in a 24 hour period. Our case definition was probably more sensitive than that used in other telephone surveys of self-reported illness. Since we did not specifically provide a definition to our telephone survey participants they might have interpreted the term diarrhoea differently from each other and from us. In addition we were unable to exclude episodes occurring less than three weeks apart, among cases in the telephone survey, and this could have inflated rate estimates, especially in the 7-day recall group.

The most interesting observation is the marked difference between the estimates based on the 7 and 28 day recall period calls with the former suggesting a standardised illness rate of almost three times that of the later. There are several possible reasons for this difference. As was shown above a large part of this difference could be explained by decline in illness reporting with time before interview. This phenomenon has been described in several studies in the recent past and has generally been put down to respondents forgetting illness with time [15]. One observation that would support this hypothesis was that a greater proportion of cases in the 28-day recall group reported contacting their GP compared to the 7-day recall group. This observation could be interpreted as indicating that people generally are better at remembering illness from a few weeks ago if it was more severe and especially if it necessitated seeking medical attention. A possible additional explanation could be that in the 7 day recall study arm people who had recently been ill were staying at home whilst they recovered and this inflated the disease estimate.

In IID1, it was suggested that one reason why incidence rates based on the retrospective study were higher was a process of “telescoping”. This meant that people who had IID outside of the defined recall period remembered this illness as being more recent [4]. It would be difficult to use this to explain the discrepancy between the 7 and 28-day recall periods given that the highest reported daily illness rates were in the 48 hours prior to interview (Fig 2). However, one aspect where telescoping may be playing a role is digit preference for days with multiples of 7 [23]. There are peaks in illness onset rates at 7, 14, 21 and 28 days prior to the interview. So it is likely that someone who was ill 6 or 8 days prior to the interview would remember that it was one week (7 days) ago inflating the rates in the 7 day recall period.

## Conclusion

We have presented estimates of the standardised rate of intestinal infectious disease in the UK using a retrospective telephone survey. However, the estimated rate is influenced strongly by the case definition used and whether or not participants are asked to recall illness over the previous 7 or 28 days. Furthermore, whatever recall period is used the resultant rate generated by this retrospective study is much greater than that found in the prospective arm of the UK IID2 study. These findings challenge the definition of incidence of IID in the community and the choice of the study design that best estimates this “true” value. It seems to us that the “true” burden of intestinal infectious disease in the community is difficult to estimate, due to the complexity in measuring it and the choice of the study design is dependent on the definition of the aspects of burden of IID that researchers aim to investigate. Retrospective telephone surveys are likely to be the most cost efficient study design for estimating whether the community incidence of IID is changing with time, despite the challenges on the choice of the recall period. A 7 day recall telephone survey in particular may offer a useful surveillance tool for detecting changing disease incidence. However, they are inappropriate for example to estimate the relative contributions of different enteric pathogens as faecal sample collection would be difficult. For this, prospective study design would be better-suited.

## Supporting Information

S1 File Supplementary tables and figures. (DOCX)
