# Supplementary material for: Estimating the Incidence of Acute Infectious Intestinal Disease in the Community in the UK: A Retrospective Telephone Survey
Source: PLoS One. 2016 Jan 25;11(1):e0146171. doi: 10.1371/journal.pone.0146171 (PMC4725772; doi:10.1371/journal.pone.0146171)
Supplement: S1 File — (DOCX) [file pone.0146171.s001.docx]

**Supplementary tables and figures**

**Table A: Percentage of eligible calls resulting in completed interviews by country**

|  |  | **Completed interviews** | **Refusals / Interviews not completed** | **Total** |
| --- | --- | --- | --- | --- |
| England | *N* | 4,059 | 3,799 | 7,858 |
|  | *% (95% CI)* | *51.7 (50.5; 52.8)* |  |  |
| Northern Ireland | *N* | 3,752 | 5,245 | 8,997 |
|  | *% (95% CI)* | *41.7 (40.7; 42.7)* |  |  |
| Scotland | *N* | 3,642 | 3,652 | 7,294 |
|  | *% (95% CI)* | *49.9 (48.8; 51.1)* |  |  |
| Wales | *N* | 4,755 | 4,817 | 9,572 |
|  | *% (95% CI)* | *49.7 (48.7; 50.7)* |  |  |
| *Total* | *N* | *16,208* |  | *33,721* |
|  | *% (95% CI)* | 48.1 (47.5; 48.6) |  |  |

**Fig A: Eligibility of calls made in the Telephone Survey, UK**

|  | | | |
| --- | --- | --- | --- |
|  |  |  |  |
| a | - 10,222 no answer - 3,693 answering machine - 2,108 fax machine - 358 engaged | b | - 24,341 invalid number - 4,395 commercial number - 40 non-English speaker |

**Fig B: Number of completed interviews by month**

**Fig C: Distribution of ethnic group among Telephone Survey participants relative to the UK census population**

**Fig D: Distribution of household size among Telephone Survey participants compared with the UK census population**

 NOTE: the percentage of participants in each category is averaged across the 4 UK countries taking into account the relative size of the population in each country

**Fig E: Distribution of area-level deprivation among Telephone Survey participants**

NOTE: the proportion of participants in each category is a weighted average that takes into account the different distribution of participants across countries.

**Fig F: Distribution of urban-rural classification among Telephone Survey participants compared with the UK census population**

 NOTE: the percentage of participants in each category is averaged across the 4 UK countries taking into account the relative size of the population in each country
